# Inhibition of glycolysis enhances the efficacy of immunotherapy via PDK-mediated upregulation of PD-L1

**DOI:** 10.1007/s00262-024-03735-0

**Published:** 2024-06-04

**Authors:** Ruizhi Zhang, Gan Mao, Yu Tang, Chong Li, Yisong Gao, Wenxiang Nie, Tianyu Song, Suao Liu, Peng Zhang, Kaixiong Tao, Wei Li

**Affiliations:** 1 Department of Gastrointestinal Surgery, Union Hospital, Tongji Medical College, Huazhong University of Science and Technology, Wuhan, China; 2 Department of Gastrointestinal Surgery, Zhejiang University School of Medicine First Affiliated Hospital, Hangzhou, China; 3 Department of Breast and Thyroid Surgery, Union Hospital, Tongji Medical College, Huazhong University of Science and Technology, Wuhan, China

**Keywords:** Stomach neoplasms, Immunotherapy, PD-L1, Glycolysis

## Abstract

**Background:**

Immunotherapy for gastric cancer remains a challenge due to its limited efficacy. Metabolic reprogramming toward glycolysis has emerged as a promising avenue for enhancing the sensitivity of tumors to immunotherapy. Pyruvate dehydrogenase kinases (PDKs) play pivotal roles in regulating glycolysis. The importance of PDKs in the context of gastric cancer immunotherapy and their potential as therapeutic targets have not been fully explored.

**Methods:**

PDK and PD-L1 expression was analyzed using data from the GSE66229 and The Cancer Genome Atlas (TCGA) cohorts. Additionally, the Immune Checkpoint Blockade Therapy Atlas (ICBatlas) database was utilized to assess PDK expression in an immune checkpoint blockade (ICB) therapy group. Subsequently, the upregulation of PD-L1 and the enhancement of anticancer effects achieved by targeting PDK were validated through in vivo and in vitro assays. The impact of PDK on histone acetylation was investigated using ChIP‒qPCR to detect changes in histone acetylation levels.

**Results:**

Our analysis revealed a notable negative correlation between PD-L1 and PDK expression. Downregulation of PDK led to a significant increase in PD-L1 expression. PDK inhibition increased histone acetylation levels by promoting acetyl-CoA generation. The augmentation of acetyl-CoA production and concurrent inhibition of histone deacetylation were found to upregulate PD-L1 expression in gastric cancer cells. Additionally, we observed a significant increase in the anticancer effect of PD-L1 antibodies following treatment with a PDK inhibitor.

**Conclusions:**

Downregulation of PDK in gastric cancer cells leads to an increase in PD-L1 expression levels, thus potentially improving the efficacy of PD-L1 immune checkpoint blockade therapy.

**Supplementary Information:**

The online version contains supplementary material available at 10.1007/s00262-024-03735-0.

## Introduction

According to Global Cancer Statistics 2020, gastric cancer is the fifth most prevalent cancer worldwide and the fourth leading cause of cancer-related deaths, with more than one million new cases reported each year [1]. Although gastric cancer patient outcomes have improved with the development of adjuvant chemoradiotherapy, overall survival (OS) rates have remained unsatisfactory [2]. However, substantial progress has been made in the treatment of various tumors via immunotherapy checkpoint inhibition. In gastric cancer, immune checkpoint blockade (ICB) therapy has shown promising efficacy and safety [3]. In 2021, the FDA approved Opdivo, a programmed cell death 1/programmed cell death ligand 1 (PD-1/PD-L1) inhibitor, for the initial treatment of patients with advanced gastric cancer [4]. Despite ongoing efforts to increase the efficacy of immunotherapy, the results in patients with gastric cancer remain unsatisfactory [5]. Therefore, investigating potential factors that modulate PD-L1 expression is critical for enhancing the effectiveness of ICB therapy.

Tumors predominantly rely on aerobic glycolysis, often referred as the Warburg effect, to satisfy their energy demands [6]. Recent research has shown that this metabolic shift can enable tumor cells to evade antitumor immunity [7, 8]. Increased tumor glycolysis disrupts T-cell function by influencing the behavior of memory and naive T cells by uptaking glucose and releasing of various byproducts in the tumor microenvironment [9, 10]. Moreover, metabolic reprogramming toward glycolysis serves as a molecular mechanism capable of modifying tumor sensitivity to immunotherapy. Importantly, tumor metabolism not only affects the activity of immune cells but also plays a regulatory role in the gene expression of tumor cells. While previous studies have investigated how tumors impact immune cell function, limited research has investigated the relationship between glycolysis and the immunoregulatory molecule PD-L1 in tumor cells [11].

The aim of this study was to evaluate the influence of glycolysis on PD-L1 expression in tumor cells, revealing a previously unelucidated mechanism. Our research revealed a correlation between the upregulation of PD-L1 expression in tumor cells and the expression of glycolysis-related genes in gastric cancer patients. In vitro experiments demonstrated that knocking down PDK (pyruvate dehydrogenase kinase) upregulated PD-L1 expression in tumor cells. Moreover, PDK inhibition enhanced the tricarboxylic acid (TCA) cycle, acetoacetyl coenzyme A (acetyl-CoA) generation, and subsequent histone acetylation, leading to the upregulation of PD-L1 expression. These findings shed light on the mechanism by which glycolysis regulates PD-L1 expression in tumor cells and may provide valuable insights for the development of innovative strategies to improve the efficacy of PD-L1-targeted therapies.

## Materials and methods

### Cell culture and reagents

The human gastric cancer cell line AGS (RRID: CVCL_0139) and the mouse gastric cancer cell line MFC (RRID: CVCL_5J48) were acquired from the National Collection of Authenticated Cell Cultures of China. The AGS and MFC cell lines were cultured in RPMI-1640 medium (Gibco) supplemented with 10% fetal bovine serum (FBS; Gibco), streptomycin (100 mg/ml) and penicillin (100 U/ml). HEK293T cells were maintained in DMEM(Gibco) supplemented with high glucose, 10% fetal bovine serum, streptomycin (100 mg/ml) and penicillin (100 U/ml), in a humidified incubator at 37 °C.

The PDK inhibitor dichloroacetate (DCA, 347,795) and the HAT inhibitor anacardic acid (ANAC, A7236) were obtained from Sigma (Sigma-Aldrich, USA). Additionally, the ACLY inhibitor BMS-303141 (BMS, SML0784) and 3-bromopyruvic acid (3-BP, 16,490) were obtained from Sigma (Sigma-Aldrich, USA).

### Short hairpin RNA (shRNA) knockdown

After PDK shRNA (short hairpin RNA) target sequence cloned into the PLKO.1 vector, the HEK293T cells were transfected with vector (4 µg) along with psPAX2 (3 µg) and pMD2.G (1 µg) helper constructs using Lipofectamine 2000 reagent (Life Technologies, Carlsbad, CA, USA). Transfection was performed by using Lipofectamine 2000 reagent (Life Technologies, Carlsbad, CA, USA) for 48 h. Retroviral supernatants were collected from the media and subsequently filtered through 45 μm filters. To generate PDK knockdown cells, MFC cells were infected with retroviral supernatants and 10 µg/ml polybrene for 48 h in a 6-well plate. The cells were cultured at 37 °C with 5% CO2. After the virus was removed, the cells were selected with 10 µg/ml puromycin (Sigma Aldrich, USA) for 48 h.

### Western blot analysis

Cultured cells were harvested by washing them with phosphate-buffered saline (PBS) twice. The collected cells were lysed using radioimmunoprecipitation assay (RIPA) buffer (Sigma Aldrich, USA, R0278). For histone extraction, whole cell lysates were prepared using lysis buffer (10 mM HEPES pH 7.4, 10 mM KCl, 0.05% NP-40) supplemented with protease and phosphatase inhibitors, including 1 mM sodium orthovanadate, as well as deacetylase inhibitors, including 5 mM TSA. The histones were extracted through centrifugation. The protein concentration was determined using the bicinchoninic acid (BCA) method (Sigma Aldrich, USA, P0012). The total protein samples were sonicated and mixed with SDS loading buffer before being boiled at 100 °C for 10 min. Subsequently, the samples were separated using 10% and 15% sodium dodecyl sulfate–polyacrylamide gel electrophoresis (SDS–PAGE). After being blocked with 5% nonfat milk for 1 h, the membrane was incubated in the antibody solution overnight at 4 °C. After three washes, the membrane was incubated with anti-rabbit secondary antibodies at room temperature for 1 h. Immunoblots were visualized using an Invitrogen iBright CL1000 imaging system (Thermo Fisher Scientific). The primary antibodies used were anti-PD-L1 (CST, 13,684, 1:1000), anti-histone H3 (CST, 4499, 1:1000), and anti- acetyl-histone H3 (Lys9) (H3K9ac) (CST, 9649, 1:1000).

### Multiplex immunohistochemical (mIHC)

FFPE sections were dewaxed and rehydrated. Antigen retrieval and endogenous peroxidase were performed using citrate buffer and 3% H_2_O_2_. The sections were blocked with immunostaining blocking solution (Proteintech, PR30008) for 1 h and then incubated with anti-PDK4 (Proteintech, 12,949–1-AP) and anti-PD-L1 (CST, 13,684) antibodies overnight. The slides were incubated with corresponding HRP-conjugated secondary antibodies and stained with DAPI.

### T cell activation

For the preparation of mouse CD8^+^ T cells, T cells were purified from C57BL/6 J mice using the mouse CD8 naïve T cell isolation kit (Biolegend, 480043). T cells were cultured in the 48-well plate precoated with 2 μg/ml anti-mouse CD3 (Biolegend, 100340) and 5 μg/ml anti-mouse CD28 (Biolegend, 102116) plus rhIL-2 (R&D, 402-ML-020) for 3 days. CD8^+^ T cells were co-cultured with tumor cells at 1:1 ratio for 24 h.

### T cell apoptosis

T cells and tumor cells were washed and stained with APC anti-mouse CD8. Subsequently, the collected cells were stained with FITC Annexcin V Apoptosis Detaction Kit with PI (Biolegend, 640914) and analyzed using a flow cytometer.

### Glucose assay and Lactate assay

For incubated cells, the cells were washed by PBS twice and sonicated. For glucose detection, the samples were mixed with GOD reagent for 10 min at 37 °C using the glucose oxidase method. For lactate detection, the sample were mixed with enzyme working solution and chromogenic agent. Vortex to mix well and incubate at 37 °C for 10 min. Add a terminator to stop the reaction. And the result were detected by MicroplateReader.

### Chromatin immunoprecipitation (ChIP)

The ChIP assay was conducted using a kit (CST, 9003) following the manufacturer's protocol. In brief, the cultured cells were crosslinked by fixing them with 1% formaldehyde for 10 min to crosslink proteins, including histone and nonhistone proteins with DNA. Subsequently, the crosslinking reaction was quenched using glycine, followed by two washes with ice-cold PBS. The cells were then subjected to incubation in buffers A and B. After micrococcal nuclease digestion and lysis in ChIP buffer, the lysate was sonicated to fragment the chromatin, which was subsequently clarified through centrifugation. A portion of the lysate was set aside as the input control. To isolate the crosslinked DNA associated with histones, immunoprecipitation was carried out by adding the target antibody (H3K9ac, CST, 9649) to the lysate, followed by overnight rotation at 4 °C. An IgG antibody (CST, 2729) was used as a negative control. Subsequently, the lysate was incubated with ChIP‒Grade Protein G Magnetic Beads while rotating for 2 h at 4 °C. Afterward, the chromatin was subjected to high-salt and low-salt wash buffers for purification. Once the crosslinks were reversed, DNA purification was performed using spin columns. The primer pairs, used for chromatin landscape mapping are listed in Supplementary Table 1 and were designed to cover regions approximately 2000 bp upstream and 1000 bp downstream of the PD-L1 transcription start site (TSS). DNA enrichment was calculated using the percent input method, defined as follows: percent input = 2% × 2 ^ (Ct 2% Input sample − Ct IP sample), where Ct represents the threshold cycle of the PCR.

### Flow cytometry

The cultured cells were collected by washing twice with PBS and then subjected to trypsin digestion. A single-cell suspension was prepared using FACS (fluorescence-activated cell sorting) buffer (2% FBS and 0.5 mM EDTA). Next, the cells were incubated with 1–2 µl of the appropriate antibodies in 100 µl of FACS buffer for 20 min at 4 °C in the dark. Afterward, the cells were washed twice with FACS buffer and then subjected to centrifugation. Subsequent analysis was performed using a flow cytometer (BD, USA). In the case of resected tumor tissues in the in vivo assay, the tissues were initially sectioned and homogenized into a single-cell suspension through a 100-μm strainer in FACS buffer. Lymphocytes were isolated using mouse lymphocyte separation medium followed by centrifugation. After centrifugation, the cells were activated for 4 h using activation medium containing 1 μg/mL ionomycin, 20 ng/ml PMA, 0.7 μL/ml Golgi stop, and 1 μL/mL Golgi plug at 37 °C. Subsequently, the cells were centrifuged, resuspended, stained with the appropriate antibodies, and analyzed using a flow cytometer. The anti-CD3, anti-CD45, anti-CD4, anti-CD8, anti-TNF-α, anti-IFN-γ, and anti-PD-L1 antibodies were purchased from BioLegend, USA.

### Quantitative real-time polymerase chain reaction (qRT‒PCR)

Total RNA was extracted from cultured cells using TRIzol reagent (Takara, 9109, Japan) and then reverse transcribed into cDNA using an RR036 kit (Takara, Japan). Subsequently, the cDNA samples were appropriately diluted with RNase-Free dH_2_O, and qRT‒PCR was conducted using TB Green Premix Ex Taq II (Takara, RR820A) on the StepOnePlus™ system. The relative gene expression and fold change were determined utilizing the comparative Ct method (2^−ΔΔCt^) and normalized to the β-actin level. The specific sequences of the primers used for qRT‒PCR are provided in Supplementary Tables 2 and 3.

### Bioinformatics analysis

Gene set enrichment analysis (GSEA) was performed to identify the underlying pathways [12, 13]. The gene set c2.all.v2023.2.Hs.symbols.gmt used for the enrichment analysis was obtained from the Molecular Signatures Database (MsigDB). The GEO: GSE66229 dataset was downloaded from the Gene Expression Omnibus (GEO) [14]. The relative expression levels of genes in gastric cancer tissues were assessed using publicly available RNA-seq data from The Cancer Genome Atlas (TCGA) database, accessed through the UALCAN portal [15]. Correlation analysis between the expression of PD-L1 and that of various PDKs in gastric cancer tissues was conducted using RNA-seq data from TCGA through the CBIOPORTAL web server [16]. The relative expression levels of PDK2 and PDK4 in gastric cancer patients receiving anti-PD-1 antibody treatment were examined utilizing publicly available RNA-seq data from the Immune Checkpoint Blockade Therapy Atlas (ICBatlas) database [17].

### Liquid chromatography‒mass spectrometry (LCMS) analysis

Cultured cells were immersed in a precooled mixture (methanol: acetonitrile: water = 2:2:1). After sonication, the sample was incubated at − 20 °C for 1 h. Protein extraction was carried out using SDT lysis buffer (2% SDS, 100 mM DTT, 100 mM Tris–HCL, pH 7.6), and the protein concentration was determined using the BCA method (Sigma-Aldrich, USA, P0012). Succinate-d6 and alanine-d4 were added, followed by vacuum drying. The analysis was conducted using the QTRAP5500 mass spectrometer (AB SCIEX, USA).

### In vivo assays

For the gastric cancer syngeneic model, 1 × 10^6^ mouse gastric cancer cells (MFCs) were subcutaneously injected into the right flank of 6-week-old male 615 mice, obtained from the Tianjin Institute of Hematology. After 1 week, the mice were randomly divided into four groups: the control group, DCA group, PD-L1 antibody group, and DCA plus PD-L1 antibody group. PD-L1 antibody, purchased from BioXCell, was administered via intraperitoneal injection at a dosage of 200 μg every 3 days. DCA was administered via intraperitoneal injection at a dosage of 100 mg/ (kg day). Bodyweight and tumor size were measured every 2 days. The tumor volume was calculated as (length × width × width)/2. The mice were euthanized after 13 days of treatment. All of the mice were housed under specific pathogen-free conditions, and the procedures were conducted following the approval of the Ethics Committee of Union Hospital, Tongji Medical College, Huazhong University of Science and Technology.

### Statistical analysis

Statistical analysis was performed using OriginPro 2022b, imageJ and GraphPad Prism 9.0. The data are presented as the mean ± standard deviation (SD), and fold changes were calculated relative to those of the control groups. For comparisons between two groups, two-tailed and unpaired Student’s t-tests were applied. To analyze the differences among multiple groups, we utilized one-way ANOVA followed by Sidak’s multiple comparison test. P < 0.05 indicated statistical significance.

## Results

### Correlation of PD-L1 expression and the ICB response with glycolysis-related genes in gastric cancer

**Fig. 1:**
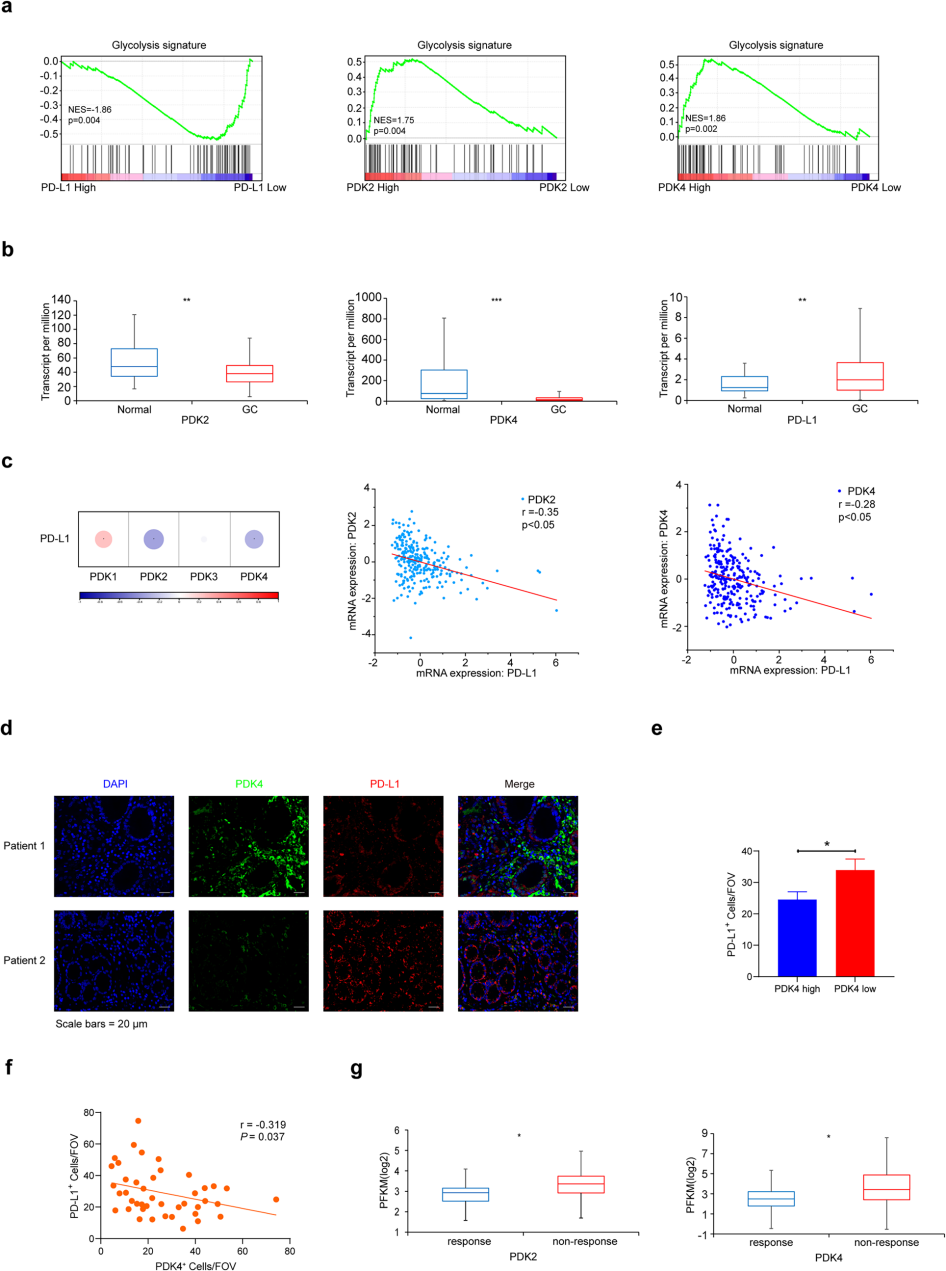
Correlation of PD-L1 Expression and the ICB Response with Glycolysis-Related Genes in Gastric Cancer. **a** Glycolysis signature analysis of PDKs and PD-L1 was conducted using the GSE66229 dataset (n = 400). **b** The comparison of PD-L1 and PDKs were performed using RNA-seq data of normal gastric tissues (n = 34) and gastric cancer tissues (n = 415) from the TCGA. **c** Spearman correlation analysis of PDKs and PD-L1 were performed using gastric cancer RNA-seq data from the TCGA (n = 295). **d** PDK4 and PD-L1 via multiplex immunohistochemical in human gastric cancer tissue. **e** PD-L1 positive cells/FOV in different PDK4 expression groups (n = 43). **f** Correlation between PDK4 and PD-L1 positive cells/FOV. **g** PDKs levels of the responders and nonresponders to PD-1 antibody treatment

By analyzing PD-L1 expression in 400 gastric cancer patients, we identified an enriched glycolysis signature in patients with low PD-L1 expression (Fig. 1a). Several key genes involved in glycolysis, including hexokinase (HK), pyruvate dehydrogenase (PDH), and lactate dehydrogenase (LDH), played important roles (Fig. 1a, Fig. S1a). To assess the impact of glycolysis-related genes on PD-L1 expression in gastric cancer, we leveraged data from the publicly available TCGA database. Our analysis revealed differential expression of glycolysis-related genes between gastric cancer tissues and normal tissues, with upregulation of PD-L1 and concurrent downregulation of HK1, PDK2, and PDK4 (Fig. 1b, Fig. S1b). The mammalian PDK family consists of four members that inhibit PDH. We also conducted a correlation analysis between PD-L1 expression and glycolysis-related gene expression in gastric cancer using RNA-seq data from the TCGA database. Our findings highlighted a significant negative correlation between PD-L1 expression and PDK2 or PDK4 (Fig. 1c). However, no significant correlation was observed between PD-L1 and HK1 (Fig. S1c). To assesse the impact of PDK on the expression of PD-L1, we conducted multiplex immunohistochemical to quantify PDK4 and PD-L1 expression in gastric cancer tissues. There was a negative correlation between PD-L1 expression and PDK4 expression (Fig. 1d-f). Furthermore, our analysis revealed an association between PD-L1 expression in gastric cancer patients and their response to ICB therapy. Specifically, low expression levels of PDK2 and PDK4 were associated with a favorable response to ICB therapy in gastric cancer patients (Fig. 1g).

### Upregulation of PD-L1 expression in gastric cancer cells through PDK inhibition

**Fig. 2:**
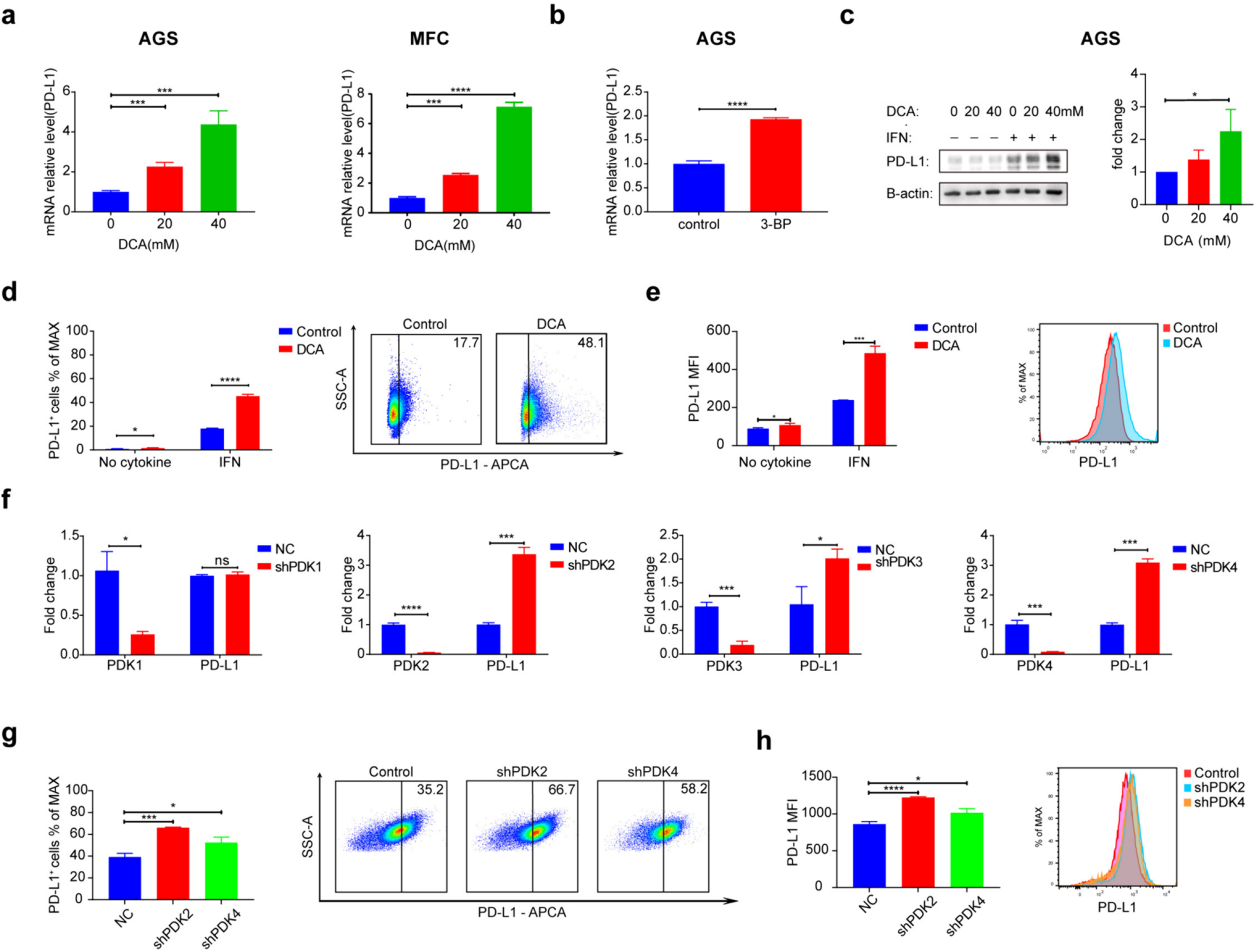
Upregulation of PD-L1 Expression in Gastric Cancer Cells through PDK Inhibition. **a** PCR analysis of PD-L1 after two gastric cancer cell lines were treated with 0 to 40 mM DCA and IFN for 24 h. Data are presented as the mean ± SD (n = 3). **b** PCR analysis of PD-L1 after the human gastric cell line AGS was treated with 3-BP and IFN for 24 h. Data are presented as the mean ± SD (n = 3). **c** Western blot analysis of the effect of DCA treatment on the expression of PD-L1 in AGS cells. Right panel shows the quantification of the PD-L1 Western blot results in treatment with IFN (n = 3). **d**, **e** Representative FACS images and statistical analysis of PD-L1^+^ cell frequencies in mouse gastric cancer line MFC treated with 0 or 40 mM DCA and treated with no cytokine or IFN for 24 h. Color histograms represent PD-L1 staining. Data are presented as the mean ± SD (n = 3). **f** PCR analysis of PDKs and PD-L1 expression in MFC cells after treated with 0.5 ng/ml IFN for 24 h after PDKs knockdown. Data are presented as the mean ± SD (n = 3). **g**, **h** Representative FACS images and statistical analysis of PD-L1^+^ cell frequencies in NC, shPDK2 and shPDK4 groups after treated with IFN for 24 h. Color histograms represent PD-L1 staining. Data are presented as the mean ± SD (n = 3)

Subsequently, we examined the impact of PDK on PD-L1 expression by subjecting gastric cancer cells to DCA and 3-BP treatments. These compounds selectively target the functions of PDK and HK. To assess the effects at both the transcriptional and translational levels, we conducted PCR, Western blot, and flow cytometry analyses (Fig. 2a–e). Moreover, we established stable cell lines with PDK1-4 knockdown. In alignment with the observations presented in Fig. 1, the expression of PD-L1 was significantly upregulated in PDK2- and PDK4-knockdown cells (Fig. 2g–h). This upregulation of PD-L1 expression was directly correlated with PDK inhibition. Furthermore, to verify the character of PD-L1, we performed co-culture experiments. The results showed that PDK inhibition of tumor cells enhances apoptosis of CD8^+^ T cells (Fig. S2a).

### Regulation of histone acetylation levels by PDK through acetyl-CoA alterations

**Fig. 3:**
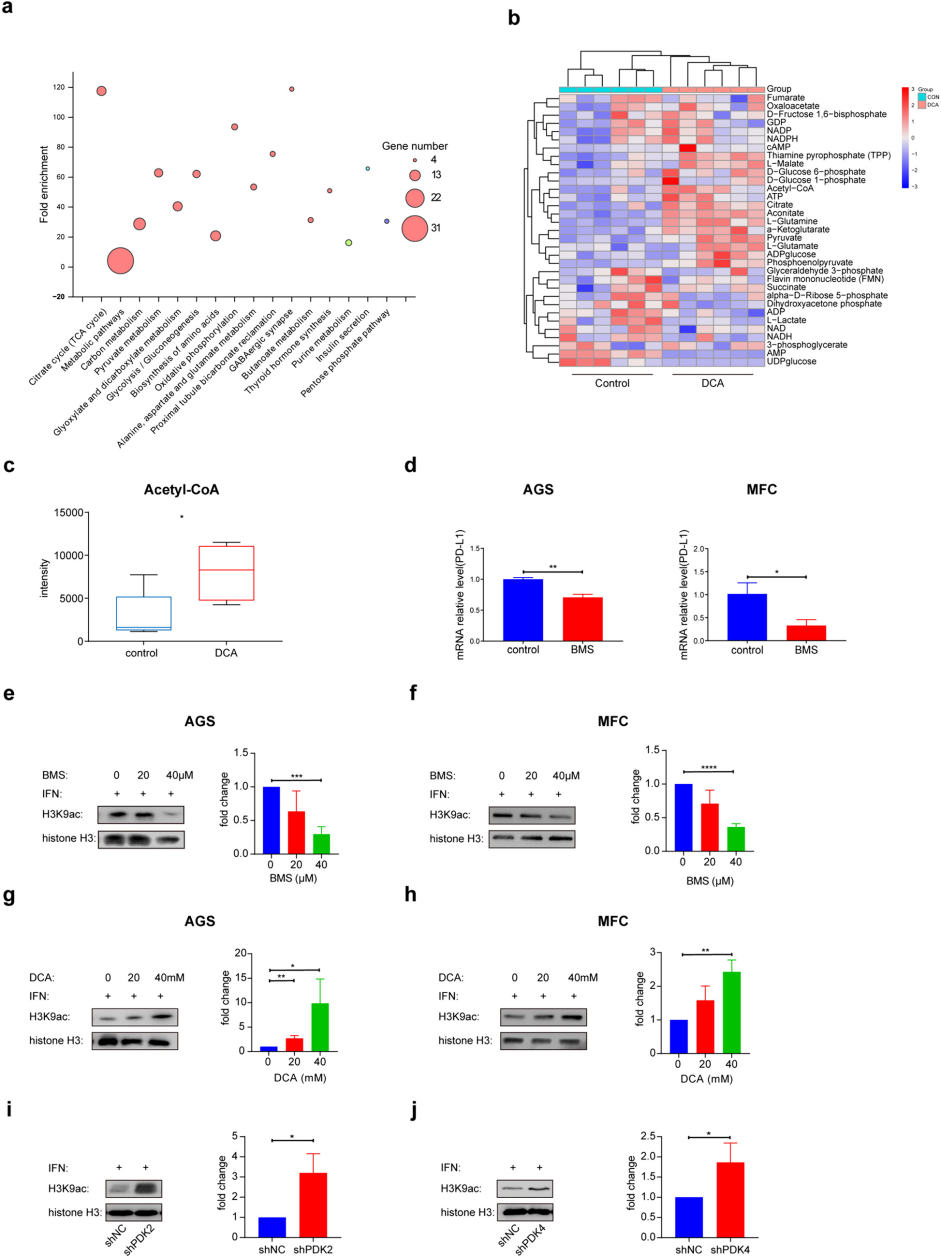
Regulation of Histone Acetylation Levels by PDK through Acetyl-CoA Alterations. **a** LCMS bubble plot of the changes in the differentially pathways after treatment with DCA. **b** Heatmap of metabolite changes in tumor cells treated with 40 mM DCA for 24 h. The red stripes in the figure represent high density, while blue stripes represent low density (n = 6). **c** Statistical analysis of acetyl-CoA in tumor cells treated with 0 or 40 mmol/L DCA for 24 h. Data are presented as min-to-max(n = 6). **d** PCR analysis of PD-L1 after gastric cancer cells treated with BMS and IFN for 24 h. Data are presented as the mean ± SD (n = 3). **e**–**f** H3 and H3K9 acetylation after AGS and MFC cells were treated with BMS and IFN for 24 h. Right panel shows the quantification of the H3K9 acetylation Western blot results (n = 3). **g**, **h** H3 and H3K9 acetylation after AGS and MFC cells were treated with DCA and IFN for 24 h. Right panel shows the quantification of the H3K9 acetylation Western blot results (n = 3). **i, j** H3 and H3K9 acetylation in shPDK2, shPDK4 and NC after treated with IFN for 24 h. Right panel shows the quantification of the H3K9 acetylation Western blot results (n = 3)

We then conducted LCMS to investigate into the pathways affected by DCA treatment. A bubble plot depicting the differentially regulated pathways revealed several significant changes, with the top three pathways being involved in the TCA cycle, metabolism, and carbon metabolism (Fig. 3a). Furthermore, to validate the specific alterations in metabolites induced by DCA treatment in MFC cells, we conducted a direct comparison of metabolite compositions between the DCA group and the control group. While the abundance of most of the metabolites associated with the TCA cycle increased in the DCA group, anaerobic glycolysis was inhibited, resulting in a significant increase in acetyl-CoA levels (Fig. 3b–c, Fig. S2b). To investigate the effects of acetyl-CoA on PD-L1 expression, we employed the small molecule ACLY inhibitor BMS-303131, which substantially reduced acetyl-CoA production. After BMS treatment, PD-L1 expression levels increased (Fig. 3d). Given the role of acetyl-CoA in histone acetylation, we examined whether inhibiting acetyl-CoA production affects histone acetylation levels. Our observations revealed that both BMS and DCA increased the level of H3K9 acetylation (Fig. 3e–h). Furthermore, when comparing H3K9 acetylation levels between the PDK knockdown group and the control group, we observed greater H3K9 acetylation in the PDK knockdown group (Fig. 3i–j). These collective findings strongly suggest that PDK regulates histone acetylation levels by controlling acetyl-CoA generation.

### Modulation of PD-L1 expression by PDK through the Influence on H3K9 acetylation

**Fig. 4:**
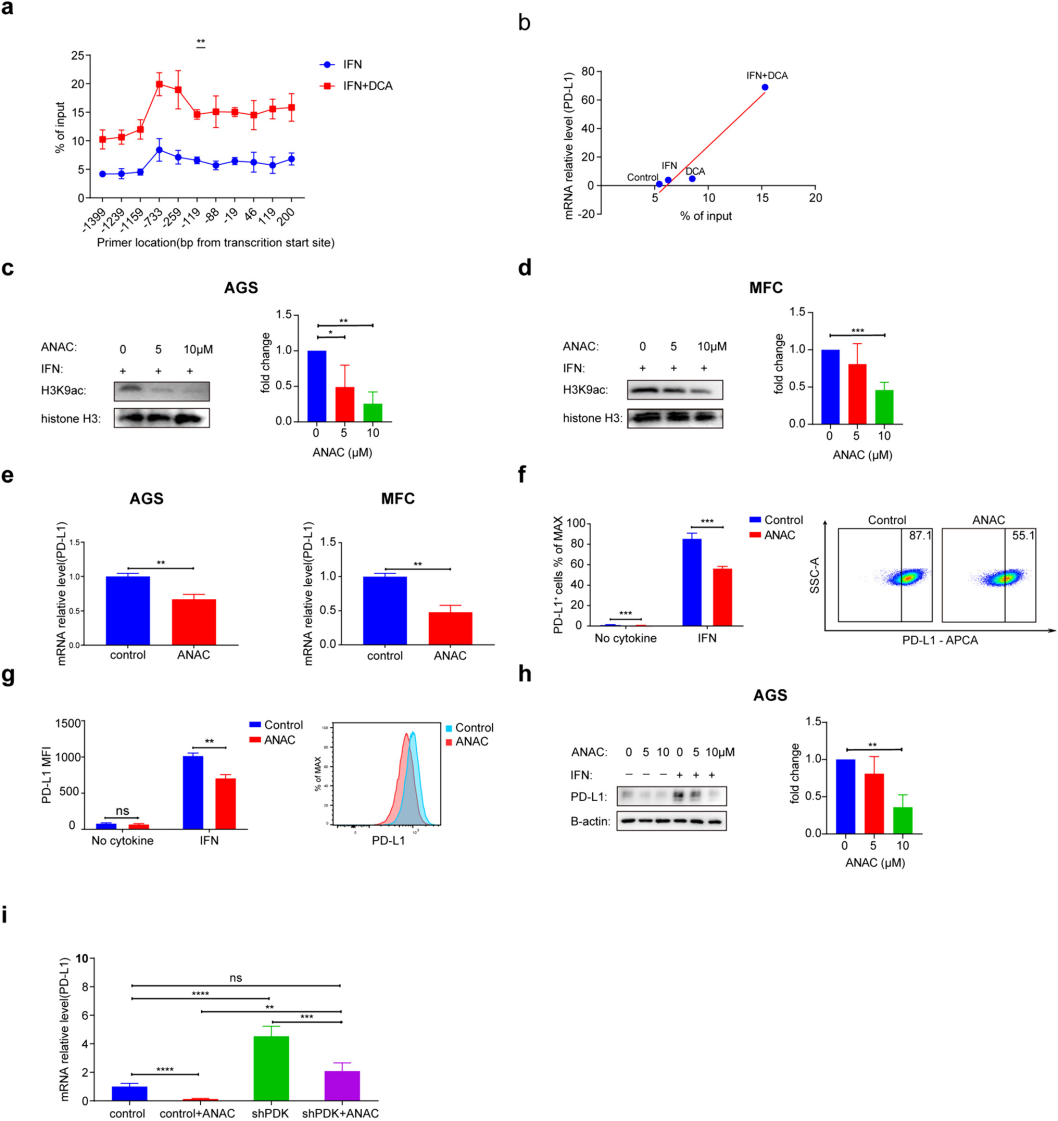
Modulation of PD-L1 Expression by PDK through the Influence on H3K9 Acetylation Levels. **a** Expression analysis of H3K9Ac enrichment in MFC cells treated with 0 or 40 mM DCA and treated with IFN for 24 h. ChIP was performed using an anti-H3K9Ac antibody, after which the immunoprecipitated PD-L1 gene promoter was quantified via PCR. **b** The correlation between enrichment of H3K9Ac on the promoter of PD-L1 and relative PD-L1 mRNA transcription in MFC cells treated with 0 or 40 mM DCA and with no cytokine or IFN for 24 h. The x-axis represent for the mean enrichment of H3K9Ac on all the promoters of the PD-L1 gene. The y-axis stood for PD-L1 mRNA expression relative to the indicated groups. **c-d** H3 and H3K9 acetylation after AGS and MFC cells were treated with ANAC and IFN for 24 h. Right panel shows the quantification of the H3K9 acetylation Western blot results (n = 3). **e** PCR analysis and western blot analysis of PD-L1 after gastric cancer cells were treated with ANAC and IFN for 24 h. Data are presented as the mean ± SD (n = 3). **f**–**g** Representative FACS images and statistical analysis of PD-L1^+^ cell frequencies in mouse gastric cancer line MFC treated with 0 or 10 µM ANAC and treated with no cytokine or IFN for 24 h. Color histograms represent PD-L1 staining. Data are presented as the mean ± SD (n = 3). **h** Western blot analysis of the effect of ANAC treatment on the expression of PD-L1 in AGS cells. Right panel shows the quantification of the PD-L1 Western blot results in treatment with IFN (n = 3). **i** PCR analysis of PD-L1 after control and shPDK cells treated with 0 or 10 µM ANAC and with IFN for 24 h

The transcription of numerous genes relies on histone acetylation and subsequent changes in chromosomal remodeling. To investigate the impact of PDK inhibition on histone acetylation at the PD-L1 gene promoter region, we conducted a ChIP‒qPCR assay to compare the enrichment of H3K9Ac on promoters after DCA treatment. H3K9Ac enrichment on promoters serves as a marker of active transcription, and we observed an increased H3K9Ac enrichment in the DCA treatment group. These findings indicated that DCA treatment led to enhanced histone acetylation at the PD-L1 gene promoter (Fig. 4a, b). To explore whether histone acetylation influenced PD-L1 expression, we used the histone acetylation inhibitor ANAC in gastric cancer cells, which resulted in a decrease in H3K9Ac enrichment (Fig. 4c, d). Subsequently, we observed PD-L1 downregulation in response to ANAC treatment (Fig. 4e–h). To determine whether PDK influences PD-L1 expression by affecting histone acetylation, we compared PD-L1 expression levels among control (NC), shPDK, and shPDK cells treated with ANAC. Notably, we observed elevated PD-L1 levels in the PDK knockdown group, but there was no significant difference between the control group and the group of shPDK cells treated with ANAC (Fig. 4i). These results indicate that PDK influences PD-L1 expression by modulating acetyl-CoA generation, thereby increasing histone acetylation. In tumor cells, PDK impacts acetyl-CoA generation via pyruvate dehydrogenase (PDH).

### Enhancing the effectiveness of ICB therapy with the PDK inhibitor DCA

**Fig. 5:**
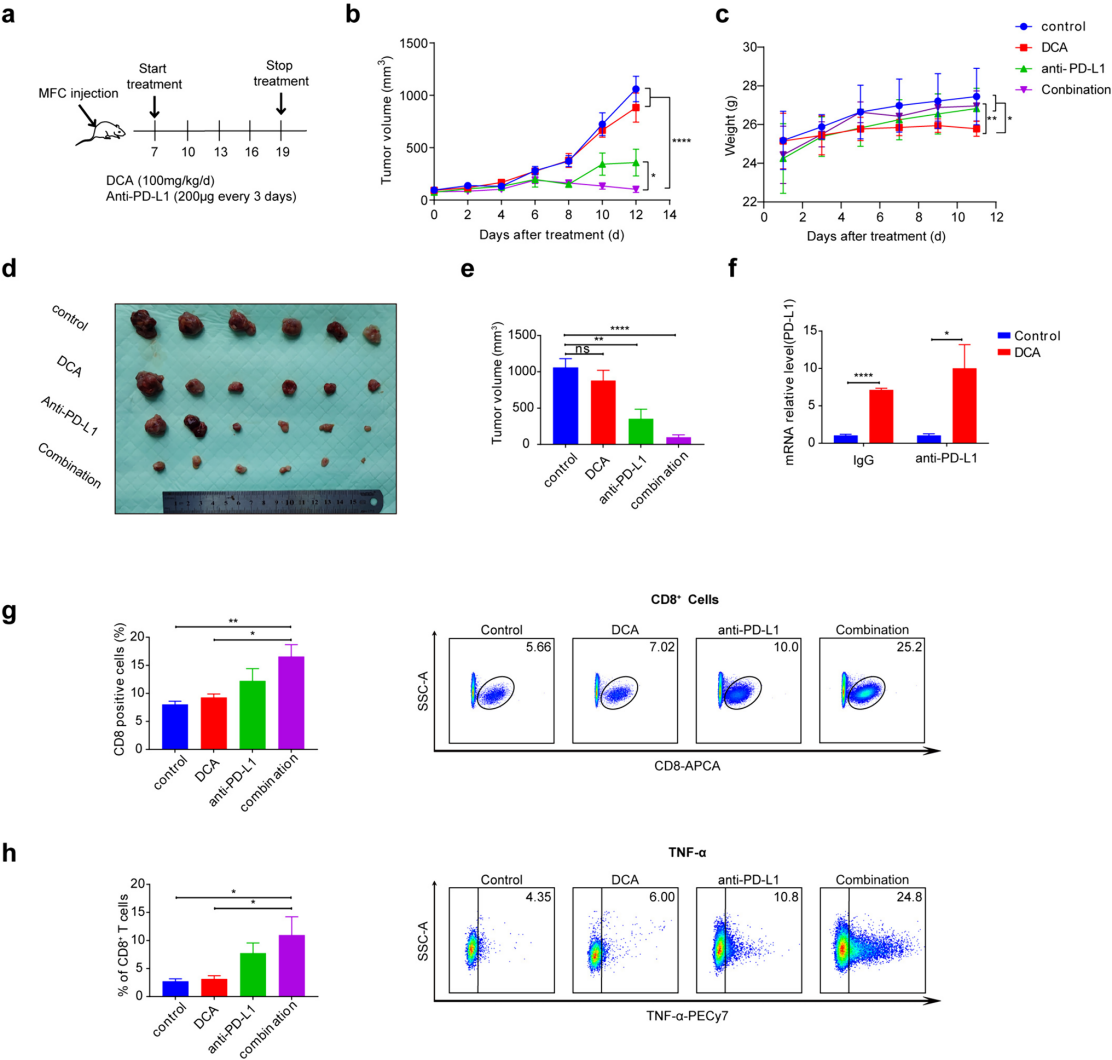
Enhancing the Effectiveness of ICB Therapy with the PDK Inhibitor DCA. **a** Schematic diagram of the isotype control IgG, DCA, anti-PD-L1 antibody and combination treatment groups. The treatments were started and stopped on day 7 and day 19 after MFC cell injection. **b** The tumor growth curves of the indicated groups. Data are presented as the mean ± SD (n = 6). **c** The weight curves of the mice in the indicated groups. Data are presented as the mean ± SD (n = 6). **d** Images of tumors from MFC gastric cancer mode mice. **e** The tumor volume in the indicated groups on day 19. Data are presented as the mean ± SD (n = 6). **f** PCR analysis of PD-L1 expression in the indicated groups. Data are presented as the mean ± SD (n = 3). **g** Representative FACS images and statistical analysis of CD8.^+^ cell frequencies in the indicated groups. The data are presented as mean ± SD (n = 6). **h** Representative FACS images and statistical analysis of TNF-α expression in the indicated groups. The data are presented as the mean ± SD(n = 6)

**Fig. 6:**
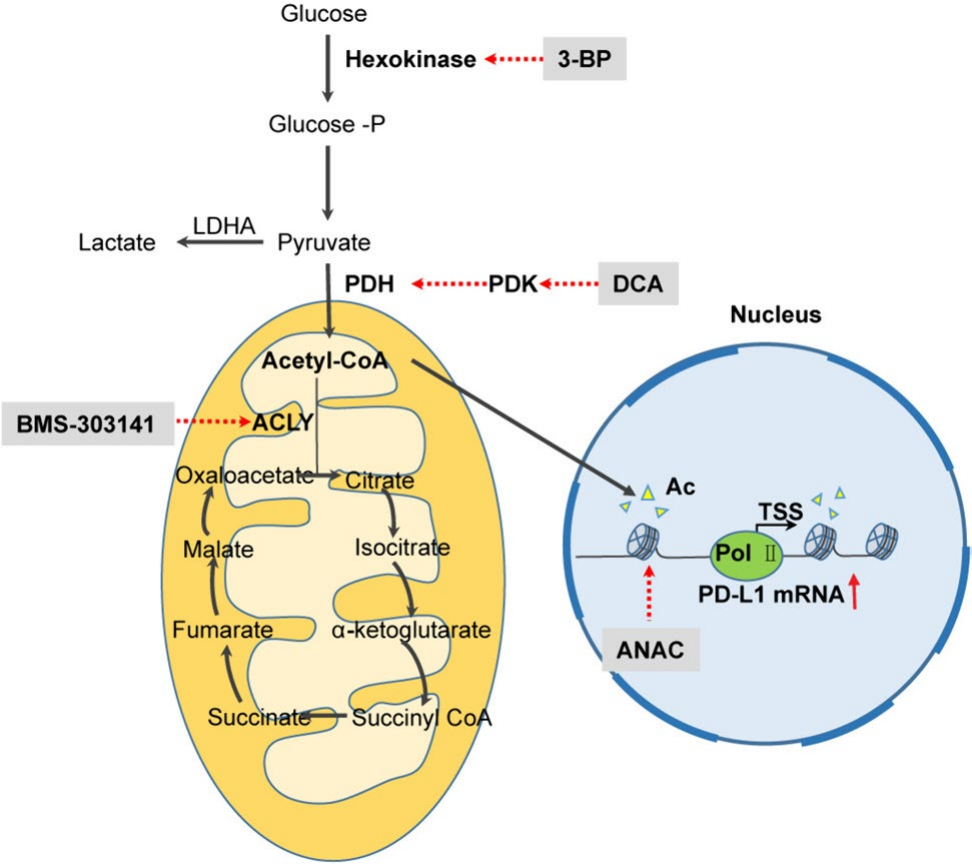
Schematic representation of the mechanism through which PDK regulates PD-L1 expression and the anticancer effect of ICB therapy in gastric cancer

Next, we investigated the potential of the PDK inhibitor DCA to augment the anticancer efficacy of anti-PD-L1 immunotherapy in vivo. Gastric cancer cells were implanted into the right flank of 7-week-old male 615 mice. 7 days after the injection of MFC cells, we initiated daily DCA treatment in conjunction with PD-L1 antibody administration every 3 days (Fig. 5a). To determine whether PDK inhibition could enhance the effectiveness of anti-PD-L1 immunotherapy, we implanted gastric tumors cells into mice and administered four doses of an anti-PD-L1 antibody (200 ug) or an isotype control concurrently with DCA treatment. Importantly, the combination treatment group exhibited a superior anticancer effect compared to the other treatment groups (Fig. 5b, d, e). We also measured the glucose and lactate in the tumor interstitial fluid. The results showed that glucose increased significantly in the DCA group and decreased lactate in the DCA group (Fig. S2c). Due to the possible long-term toxicity of DCA, we monitored the body weight of the mice, but did not observe a large decrease in body weight (Fig. 5c). Furthermore, compared with that in the control group, PD-L1 expression in the DCA treatment group was elevated under both IgG and anti-PD-L1 antibody treatment conditions (Fig. 5f). FACS analysis (Fig. 5g, h) demonstrated that, in comparison to those in the other groups, the combination treatment group exhibited significantly increased CD8^+^ T-cell infiltration and restored T-cell cytolytic function, including enhanced TNF-α production. In conclusion, our study underscores the influence of tumor cell PDK expression on PD-L1 expression and highlights its role in enhancing the anticancer effect of ICB therapy. In summary, an increase in acetyl-CoA levels upregulates PD-L1 expression by promoting histone acetylation, which in turn affects the transcription initiation factor of PD-L1 (Fig. 6).

## Discussion

Immune checkpoint blockade therapy has demonstrated therapeutic efficacy in various tumor types. However, the efficacy of PD-L1 antibodies in gastric cancer has been limited, and the underlying mechanism has yet to be studied. Previous studies have consistently indicated that patients with upregulated PD-L1 expression tend to exhibit a more favorable response to PD-L1 antibodies and prolonged OS following ICB therapy [18, 19]. Furthermore, the effectiveness of ICB can be significantly augmented when PD-L1 expression in tumor cells is upregulated [20]. This study provides novel insight showing that inhibiting the glycolysis pathway in tumor cells through PDK inhibition upregulates PD-L1 expression and enhances the anticancer effect of ICB mediated by PD-L1 antibodies.

Tumor cells undergo metabolic reprogramming to facilitate uncontrolled proliferation and reshape the tumor microenvironment [21, 22]. Our study revealed a significant negative correlation between the expression of glycolysis-related genes(PDK and HK) and PD-L1 expression in gastric cancer. These findings highlighted the impact of tumor aerobic glycolysis on the expression of crucial immune checkpoint molecules. Additionally, several studies have demonstrated that tumor metabolic processes influence the expression of immune checkpoint molecules. For example, enhanced fatty acid metabolism upregulates the expression of the immune checkpoint protein CD47, which enhances tumor immune evasion [23], and Huang et al. reported that tumor derived lactate promoted histone lactylation to induce PD-L1 transcription, leading to an immunosuppressive response in leukemia patients [24]. However, Huang’s report indicated that tumor glycolysis upregulates PD-L1 expression which contradicts our findings. The unique context of solid tumors such as gastric cancer may contribute to these distinct findings.

Restraining glycolysis leads to a shift from lactic acid production to acetyl-CoA generation. In this study, we revealed that PDK inhibition promoting the generation of acetyl-CoA. Acetyl-CoA, a pivotal substrate of the TCA cycle, serves as a signaling metabolite and plays a crucial role in histone acetylation through posttranslational lysine acetylation. Histone acetylation is a fundamental mechanism involved in epigenetic regulation and has a substantial impact on the transcriptional regulation of gene expression [25]. It facilitates the binding of transcription factors to DNA, thereby promoting the transcriptional regulation of genes [26]. Our previous study revealed that histone acetylation can influence the expression of PD-L1 [27]. Overall, these findings suggest that elevated acetyl-CoA levels induced by PDK inhibition may upregulate PD-L1 expression in gastric cancer cells. Our data confirmed that the PDK inhibitor DCA increased H3K9Ac level and promoted PD-L1 transcription.

The efficacy of PD-L1 antibodies treatment in melanoma and colorectal cancer has been demonstrated by several published studies [28, 29]. A phase I study of a novel anti-PD-L1 antibody revealed promising safety and efficacy in patients with several other advanced solid tumors [30]. However, enhancing the effectiveness of PD-L1 immunotherapy in treating gastric cancer patients remains a challenge. In this study, we observed that impeding tumor aerobic glycolysis by inhibiting PDK increased the efficacy of PD-L1 immunotherapy. Cappellesso et al. [31] analyzed single-cell RNA sequencing data in patients with pancreatic ductal adenocarcinoma, showing that inhibiting SCL4A4 mitigated acidosis in the tumor microenvironment, reduced tumor glycolysis and enhanced immunotherapy efficacy. Furthermore, alterations in tumor fatty acid metabolism promote tumor death and increase the effectiveness of PD-L1 immunotherapy [32]. Together with the findings of other studies, our study collectively suggests that modulating tumor metabolism could enhance the effectiveness of ICB therapy.

In summary, our research has illuminated the pivotal role of PDK in regulating the expression of the immune-associated molecule PD-L1 within tumor cells. The glycolytic activity of tumor cells significantly influences the expression of immune-related molecules. Our study demonstrated that glycolysis enhances histone acetylation by elevating acetyl-CoA levels and that PDK can modulate PD-L1 expression through the regulation of histone acetylation. Furthermore, our research emphasized that inhibiting PDK substantially enhances the anticancer efficacy of PD-L1 antibodies. Consequently, our findings suggest that targeting PDK may constitute a promising strategy for augmenting the efficacy of ICB therapy with PD-L1 antibodies, offering potential benefits for patients with advanced gastric cancer.

## Supplementary Information

Below is the link to the electronic supplementary material.

Supplementary file1 (DOCX 492 KB)

Supplementary file2 (DOCX 28 KB)

## Data Availability

Public datasets used in the study could be downloaded in online repositories. The original data presented in the study can be found in the article/supplementary material.

## Abbreviations

Acetyl-CoA: Acetoacetyl coenzyme A
BCA: Bicinchoninic acid assay
ChIP: Chromatin immunoprecipitation
CTLA-4: Cytotoxic T lymphocyte-associated antigen-4
DCA: Dichloroacetate
FACS: Fluorescence-activated cell sorting
FDA: Food and drug administration
GSEA: Gene set enrichment analysis
HK: Hexokinase
H3K9ac: Acetyl-histone H3 (Lys9)
ICBatlas: Immune checkpoint blockade therapy atlas
ICB: Immune checkpoint blockade
LCMS: Liquid chromatography**‒**mass spectrometry
LDH: Lactate dehydrogenase
PDH: Pyruvate dehydrogenase
PDK: Pyruvate dehydrogenase kinases
PD-1: Programmed cell death 1
PD-L1: Programmed cell death-ligand 1
qRT‑PCR: Quantitative real-time polymerase chain reaction
RIPA: Radioimmunoprecipitation assay
SDS-PAGE: Sodium dodecyl sulfate–polyacrylamide gel electrophoresis
shRNA: Short hairpin RNA
TCA: Tricarboxylic acid
TCGA: The Cancer Genome Atlas
