# Supplementary material for: Inhibition of glycolysis enhances the efficacy of immunotherapy via PDK-mediated upregulation of PD-L1
Source: Cancer Immunol Immunother. 2024 Jun 4;73(8):151. doi: 10.1007/s00262-024-03735-0 (PMC11150234; doi:10.1007/s00262-024-03735-0)
Supplement: Supplementary file 1 — Supplementary file1 (DOCX 492 KB) [file 262_2024_3735_MOESM1_ESM.docx]

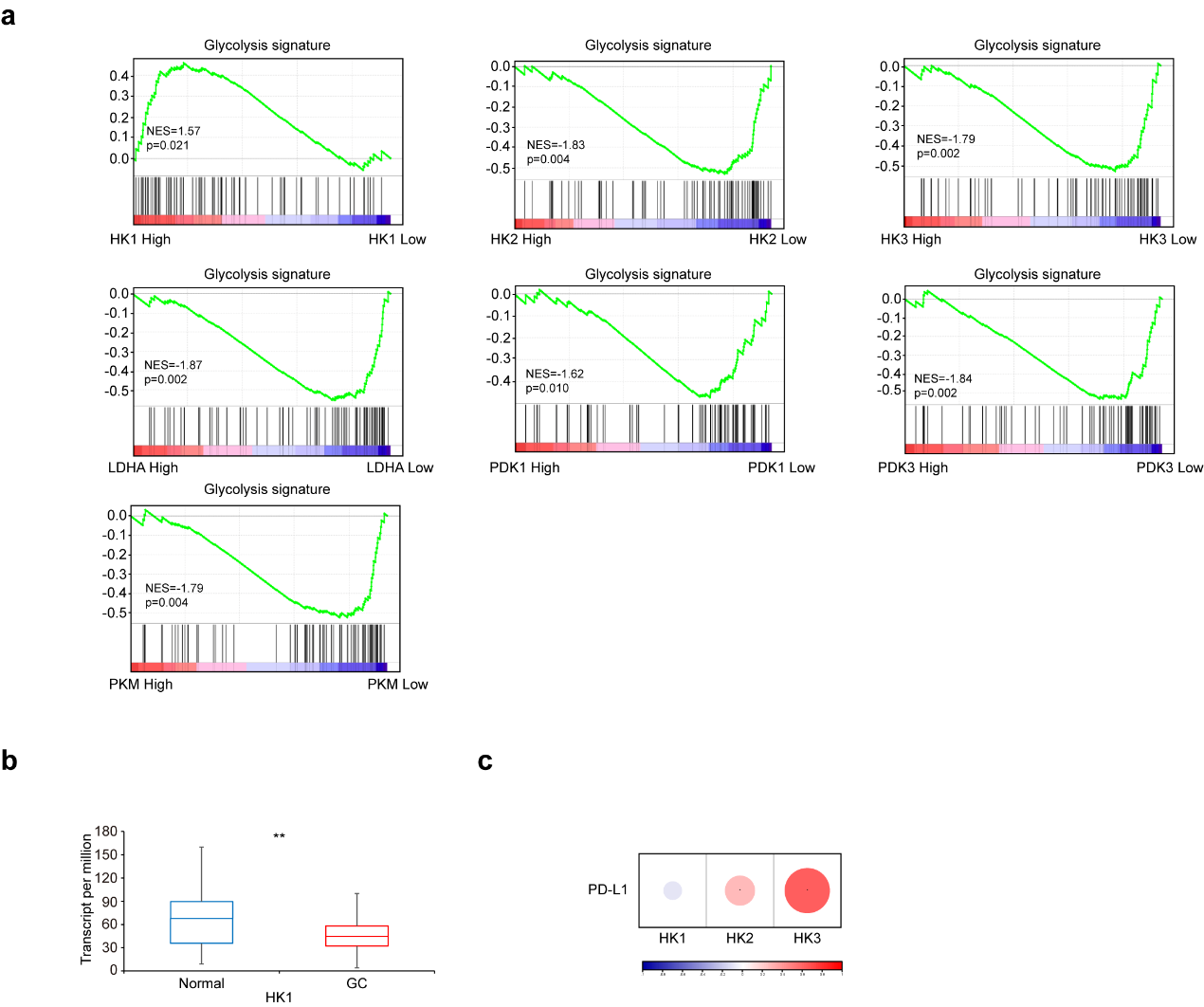


**Figure. S1. Correlation of PD-L1 Expression with Glycolysis-Related Genes in Gastric Cancer.** (a) Glycolysis signatures analysis of HKs, LDHA, PDK1, PDK3, and PKM were conducted using GSE66229 dataset (n=400). (b) The comparison of PD-L1 and HK1 were performed using RNA-seq data of normal gastric tissues (n = 34) and gastric cancer tissues (n = 415) from TCGA. (c) Spearman correlation analysis of HKs and PD-L1 were performed using gastric cancer RNA-seq data of TCGA (n=295).


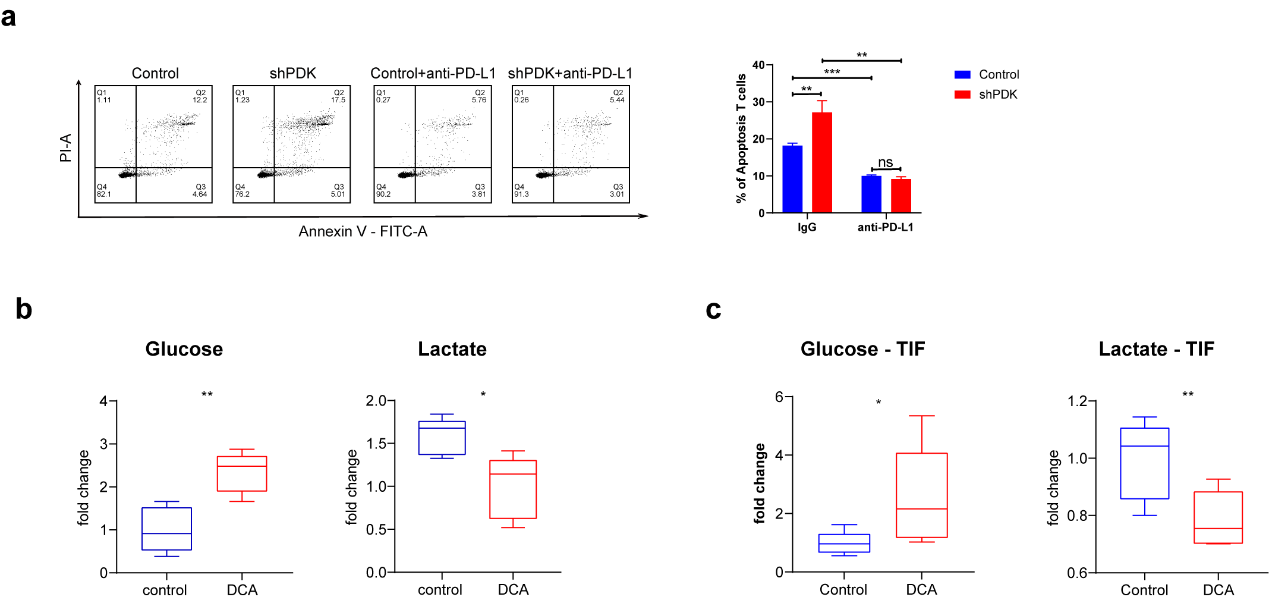
**Figure. S2. Influence of PDK Inhibition in Gastric Cancer Cells.** (a) The apoptosis of CD8^+^ T cells after coincubated with NC and shPDK cells for 24h . (b) Statistical analysis of glucose and lactate in tumor cells treated with 0 or 40 mmol/L DCA for 24 h. Data are presented as min-to-max. (c) Statistical analysis of glucose and lactate in TIF treated with DCA. Data are presented as min-to-max.
