# Supplementary material for: Inhibition of glycolysis enhances the efficacy of immunotherapy via PDK-mediated upregulation of PD-L1
Source: Cancer Immunol Immunother. 2024 Jun 4;73(8):151. doi: 10.1007/s00262-024-03735-0 (PMC11150234; doi:10.1007/s00262-024-03735-0)
Supplement: Supplementary file 2 — Supplementary file2 (DOCX 28 KB) [file 262_2024_3735_MOESM2_ESM.docx]

**Supplemental Tables**

Table 1. CHIP ‒ PCR primer for PD-L1

| Location from TSS (bp) | Forward primer (5' to 3') | Reverse primer (5' to 3') |
| --- | --- | --- |
| -1399 | GCAGCATCAACCCCAATGAC | GGGTGAATACTGGGGTGTCC |
| -1239 | AGGCCCTTGAAGTCCAACAG | GAACCGTGCCCTTTGTGAAC |
| -1159 | CCACACTTCCAGTTCGCAGA | TGCCGTTCAAGAACTTCCCA |
| -733 | GCTTCTAAAGCGCTCACTGC | CCAGGGCTAGGGTCTCAGAA |
| -259 | CAGAGACTCACCTGCCACTG | CACCCAGTCCCTTTGAGCAT |
| -119 | ACTCATGCTCAAAGGGACTGG | CGAGAAGATGCTCCCTTACCT |
| -88 | TTTCACAGACAGCGGAGGTT | TTAAAGTGCCCTGCAAGCGG |
| -19 | CAGGAAATCGTGGTCCCCAA | TTTAAAGTGCCCTGCAAGCG |
| 46 | CTGCAGGTAAGGGAGCATCT | ACCCAGCTACCTACCCACAA |
| 119 | TTTAGGACGGAGAAGGGAACC | GACCTCCGGAGATGCTTCAA |
| 200 | GGGTAGGTAGCTGGGTCAGA | TCCGGAGATGCTTCAAGTGG |

Table 2. qRT-PCR primer for mouse mRNA analysis

| gene name | Forward primer (5' to 3') | Reverse primer (5' to 3') |
| --- | --- | --- |
| PDK1 | CCACTGAGGAAGATCGACAGAC | AGAGGCGTGATATGGGCAATCC |
| PDK2 | CGCCTATGACATGGCTAAGCTC | ACAGGTGGGAAGGGACATAGAC |
| PDK3 | CCGTCGCCACTGTCTATCAAAC | CTCTCATGGTGTTAGCCAGTCG |
| PDK4 | GTCGAGCATCAAGAAAACCGTCC | GCGGTCAGTAATCCTCAGAGGA |
| PD-L1 | TGCGGACTACAAGCGAATCACG | CTCAGCTTCTGGATAACCCTCG |
| β-actin | CATTGCTGACAGGATGCAGAAGG | TGCTGGAAGGTGGACAGTGAGG |

Table 3. qRT-PCR primer for mouse mRNA analysis

| gene name | Forward primer (5' to 3') | Reverse primer (5' to 3') |
| --- | --- | --- |
| PD-L1 | TGCCGACTACAAGCGAATTACTG | CTGCTTGTCCAGATGACTTCGG |
| β-actin | CACCATTGGCAATGAGCGGTTC | CACCATTGGCAATGAGCGGTTC |
